# Application of methylene blue combined with ropivacaine intercostal nerve block in postoperative analgesia of autologous costal cartilage augmentation rhinoplasty

**DOI:** 10.1007/s00101-022-01222-8

**Published:** 2022-11-21

**Authors:** Jiang Guoyu, Wang Tao, You Xi

**Affiliations:** 1 Department of Anesthesiology, Chongqing Huamei Plastic Surgery Hospital, 400010 Chongqing, China; 2 Department of Cosmetology, Chongqing Huamei Plastic Surgery Hospital, 400010 Chongqing, China

**Keywords:** Methylene blue, Ropivacaine, Intercostal nerve block, Autologous costal cartilage comprehensive augmentation rhinoplasty, Postoperative analgesia, Methylenblau, Ropivacain, Interkostalnervenblockade, Augmentationsrhinoplastik mit autologem Rippenknorpel, Postoperative Analgesie

## Abstract

**Objective:**

To observe the effect of methylene blue combined with ropivacaine intercostal nerve block on postoperative analgesia after autologous costal cartilage augmentation rhinoplasty.

**Methods:**

In this study 100 female patients who underwent autologous costal cartilage comprehensive augmentation rhinoplasty in Chongqing Huamei Plastic Surgery Hospital from April to November 2021 were randomly divided into an experimental group and a control group, with 50 cases in each group. In the experimental group methylene blue was combined with ropivacaine intercostal nerve block as patient controlled intravenous analgesia (PCIA), and the control group was ropivacaine intercostal nerve block combined with PCIA. The visual analogue scale (VAS) scores of resting and coughing at 6 h (T1), 24 h (T2), 48 h (T3), 72 h (T4) after surgery were recorded and evaluated. At the same time, the number and times of oral analgesics were recorded as well as nausea, vomiting, burning pain and paresthesia.

**Results:**

The VAS scores of the experimental group were lower than those of the control group at all time points. At 6 h, 24 h and 48 h after surgery, the VAS score of the experimental group was lower than that in the control group, but the difference was not statistically significant (*P* > 0.05). The VAS score of calm 72 h after surgery in the experimental group was significantly lower than that in the control group (*P* < 0.05). The analgesic effect of the two groups was better when they coughed after surgery. At 6 h after surgery, the VAS score of coughing in the experimental group was lower than that in the control group, but the difference was not statistically significant (*P* > 0.05); At 24 h, 48 h and 72 h after surgery, the VAS score of the coughing state in the experimental group was significantly lower than that in the control group (*P* < 0.05).

**Conclusion:**

Intercostal nerve block with methylene blue combined with ropivacaine can achieve good postoperative analgesic effects in augmentation rhinoplasty with autologous costal cartilage

**Supplementary Information:**

The online version of this paper (10.1007/s00101-022-01222-8) contains the questionnaire CONSORT—Guidelines for publishing random control trials.

## Abstract (de)

**Ziel:**

Beobachtung der Wirkung von Methylenblau in Kombination mit einer Interkostalnervenblockade mit Ropivacain auf die postoperative Analgesie nach Augmentationsrhinoplastik mit autologem Rippenknorpel.

**Methoden:**

In dieser Studie wurden 100 Patientinnen, die sich im Krankenhaus für Plastische Chirurgie Chongqing Huamei von April bis November 2021 einer umfassenden Augmentationsrhinoplastik des autologen Rippenknorpels unterzogen hatten, nach dem Zufallsprinzip in eine Versuchs- und eine Kontrollgruppe eingeteilt, wobei jede Gruppe 50 Fälle umfasste. In der Versuchsgruppe wurde Methylenblau mit der interkostalen Ropivacain-Nervenblockade kombiniert als patientengesteuerte intravenöse Analgesie (PCIA), in der Kontrollgruppe wurde die interkostale Ropivacain-Nervenblockade mit PCIA kombiniert. Die Werte der visuellen Analogskala (VAS) für Ruhe und Husten 6 h (T1), 24 h (T2), 48 h (T3), 72 h (T4) nach der Operation wurden erfasst und ausgewertet. Gleichzeitig wurden die Anzahl und die Zeiten der oralen Analgetika sowie Übelkeit, Erbrechen, brennende Schmerzen und Parästhesie aufgezeichnet.

**Ergebnisse:**

Die VAS-Werte der Versuchsgruppe waren zu allen Zeitpunkten niedriger als die der Kontrollgruppe. Bei 6 h, 24 h und 48 h nach der Operation war der VAS-Wert der Versuchsgruppe niedriger als in der Kontrollgruppe, aber der Unterschied war statistisch nicht signifikant (*p* > 0,05); Der VAS-Wert für Ruhe 72 h nach Operation war in der Versuchsgruppe signifikant niedriger als in der Kontrollgruppe (*p* > 0,05). Die analgetische Wirkung der beiden Gruppen war besser, wenn die Patientinnen nach der Operation husteten. Sechs Stunden nach der Operation war der VAS-Wert für Husten in der Versuchsgruppe niedriger als in der Kontrollgruppe, aber der Unterschied war statistisch nicht signifikant (*p* > 0,05). Der VAS-Wert für Husten 24 h, 48 h und 72 h nach der Operation war in der experimentellen Gruppe signifikant niedriger als in der Kontrollgruppe (*p* > 0,05).

**Schlussfolgerung:**

Die interkostale Nervenblockade mit Methylenblau in Kombination mit Ropivacain kann gute postoperative analgetische Wirkungen bei Augmentationsrhinoplastiken mit autologem Rippenknorpel erzielen.

Nasal plastic surgery is the third largest cosmetic plastic surgery after breast augmentation and liposuction [1]. The demand for cosmetic and plastic surgery is steadily increasing all over the world. With the continuous improvement of people’s living standards and a large population base, China has become the country with the largest amount of plastic surgery in the world. Rhinoplasty can change the size of the nose, change the appearance of the bridge of the nose, reduce the extension of the nasal wing, and make the middle of the face have a three-dimensional feeling. Studies have shown that [2] autologous costal cartilage is simple to obtain, can be carved according to the nasal shape characteristics of patients, meet the needs of nasal plastic surgery, and has no rejection and strong support. It has a good effect in nasal plastic surgery and can be widely used in nasal plastic surgery.

Clinical observation found that [3] the incision pain after costal cartilage removal was severe, which affected the recovery and safety of patients. Patient controlled intravenous analgesia (PCIA) can alleviate some of the postoperative pain but a large dose of opioids will be accompanied by a series of adverse reactions, such as nausea, vomiting and sleepiness, which limits the use and effect of postoperative analgesia to a certain extent [4]. Methylene blue is widely used as a stain in clinical applications. Later, it was used in anorectal surgery and the treatment of discogenic low back pain. It was found that it can alleviate pain [5, 6]. In order to explore the analgesic effect of methylene blue, this study used methylene blue combined with ropivacaine nerve block in rhinoplasty and removal of autologous costal cartilage, in order to reduce the postoperative pain and improve the safety of the operation.

## Data and methods

### General data

In this study 100 female patients who underwent comprehensive augmentation rhinoplasty with autologous costal cartilage in Chongqing Huamei Plastic Surgery Hospital from April to November 2021 were selected. They were rated I–II by the American Society of Anesthesiologists (ASA) grading, aged 18–40 years, body mass 40–67 kg and body mass index (BMI) 18–23. There was no significant difference in age, BMI and other general data between the two groups (*P* > 0.05). The patients were randomly divided into experimental group and control group with 50 cases in each group. The experimental group was methylene blue combined with ropivacaine intercostal nerve block combined with PCIA group, and the control group was ropivacaine intercostal nerve block combined with PCIA group. Inclusion criteria: patients who met the requirements of autologous costal cartilage comprehensive augmentation rhinoplasty. Exclusion criteria: patients with cardiovascular and cerebrovascular diseases, serious central or peripheral nervous system diseases, liver and kidney dysfunction and other diseases, allergic to amide local anesthetics and methylene blue; calcification of costal cartilage in physical examination; abuse of psychotropic substances or long-term heavy drinking; pregnant or lactating women. This study was approved by the hospital medical ethics committee. All enrolled patients were informed and signed informed consent. A CONSORT checklist and a CONSORT flowchart are also available, which display the progress of all participants through the trial, as seen in (see supplementary information, Fig. 1).

**Fig. 1:**
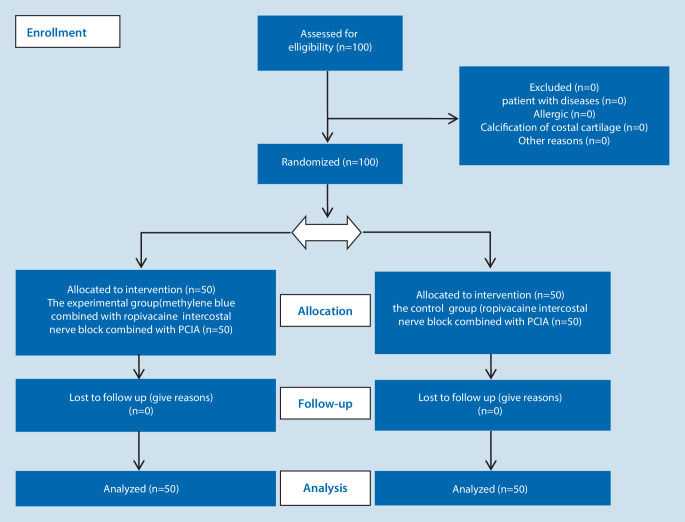
The CONSORT flowchart

### Methods

#### Anesthesia induction and maintenance

Patients in the two groups underwent routine preoperative preparation and venous access after admission. After entering the operating room, routine vital signs were monitored, and rapid anesthesia induction was performed after joint verification by the nurse, the anesthesiologist and the surgeon. Anesthesia induction drugs: midazolam 0.04 mg/kg, sufentanil 0.4 µg/kg, vecuronium 1–2 mg/kg, propofol 2 mg/kg. Endotracheal intubation was performed under visual laryngoscope, and mechanical ventilation was performed after good alignment. During the operation, propofol 4–12 mg/kg/h, remifentanil 0.05–0.20 µg/kg/min and dexmedetomidine 0.6–0.8 µg/kg/h were continuously infused to maintain the depth of anesthesia. After the costal cartilage was taken out, sufentanil 5–10 µg was given to enhance analgesia. During the operation, tropisetron 2 mg and metoclopramide hydrochloride injection 10 mg were used to stop vomiting.

#### Analgesic methods

**Fig. 2:**
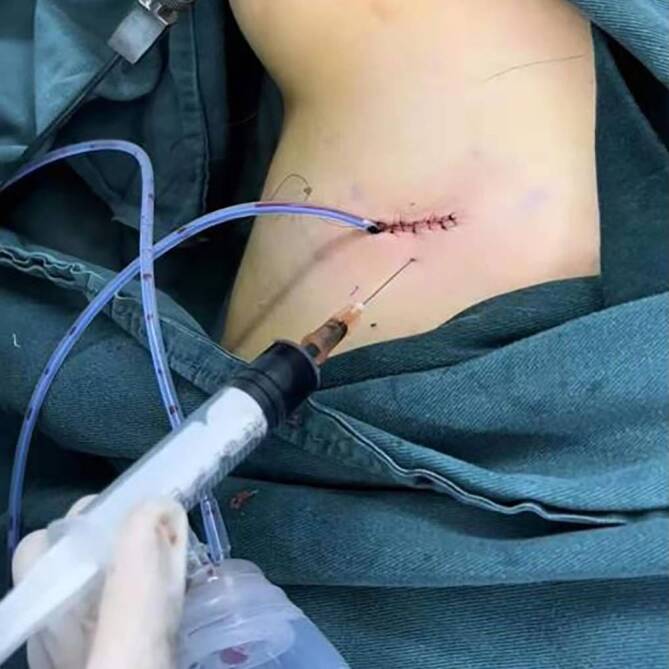
Intercostal nerve block with methylene blue and ropivacaine compound solution

Postoperative patient-controlled intravenous analgesia (PCIA) was used in both groups. Drug preparation in the pump: sufentanil 1 µg/kg plus normal saline to 100 ml. The method of continuous infusion plus single administration was adopted. The dose of continuous infusion was 2.0 ml/h, the dose of single administration was 0.5 ml, the locking time was 15 min, and the continuous analgesic time was about 48 h. Intercostal nerve block was performed by the surgeon after suturing the skin of costal cartilage area between the intercostal cartilage and the upper and lower intercostals in both groups. They were separated into two groups with a random and double blinded method, the control group and the experimental group, each group had 50 cases. In the control group, 1 ml of 0.375% ropivacaine was injected into each intercostal space. The experimental group underwent intercostal nerve block with methylene blue combined with ropivacaine (Fig. 2). Preparation of methylene blue composite solution 0.5 ml of 1% methylene blue injection into 9.5 ml of 0.375% ropivacaine sodium chloride solution to prepare 10 ml [7]. Record the number and times of taking painkillers orally (ibuprofen sustained release capsule is used for painkillers and one tablet is taken orally).

#### Observation indexes

The doctors performing the experiment recorded the pain state in two groups at calm state and cough state at 6 h (T1), 24 h (T2), 48 h (T3), 72 h (T4) after operation, and scored it with a visual analog scale (VAS). The comprehensive judgment basis of VAS score in this experiment: facial expression, patient behavior and patient expression. VAS evaluation criteria: a 10 cm horizontal line, 0 at one end indicates no pain, 10 at the other end indicates severe pain, and the middle part indicates different degrees of pain, 1–3 points for mild pain, 4–6 points for moderate pain and 7–10 points for severe pain. Record the number and times of oral analgesics as well as nausea, vomiting, burning pain and paresthesia.

### Statistical analysis

SPSS (Statistical Product Service Solutions, IBM) 22.0 statistical software was used for statistical analysis, and the measurement data were expressed. The aim of this experiment for the patients of autologous costal cartilage augmentation rhinoplasty is to improve the comfort level (VAS < 3). One way ANOVA and LSD‑t test were used for the comparison between the two groups. Two tests were used to compare the count data between groups, and the difference was statistically significant (*P* < 0.05). We calculated that group sample sizes of 20 patients (10 in group 1; 10 in group 2) would provide 80% power to reject the null hypothesis of equal means when the mean difference is −0.82 (2.16−2.98) with standard deviations of 0.61 for group 1 and 0.69 for group 2 at a two-sided alpha of 0.05. Given an anticipated dropout rate of 0%, total sample size required is 20 (10 in group 1; 10 in group 2).

## Results

**Fig. 3:**
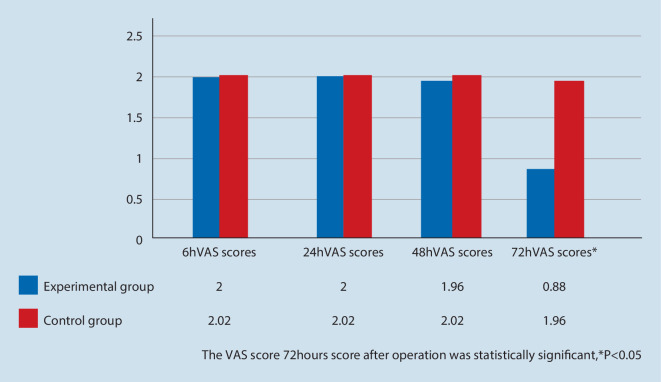
Comparison of VAS scores of calm state between the two groups at different time points after operation

In comparison with the pure postoperative PCIA analgesia technique, both groups of patients could achieve better analgesic effect in the early postoperative period. The VAS score of calm 72 h after operation in the experimental group was significantly lower than that in the control group (*P* < 0.05). as shown in Fig. 3.

**Fig. 4:**
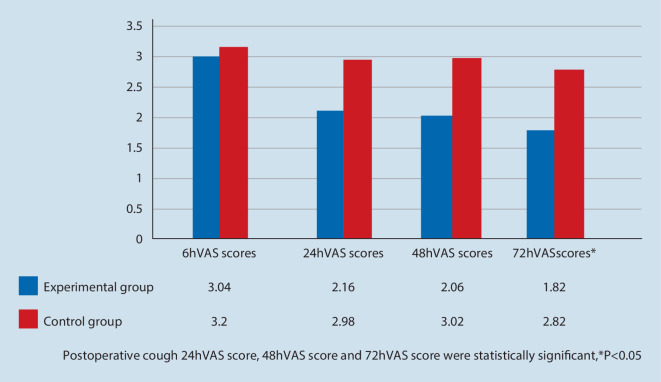
Comparison of VAS scores of cough state between the two groups at different time points after operation

In comparison with pure postoperative PCIA analgesia technique, the analgesic effect of the two groups was better. At 24 h, 48 h and 72 h after operation, the VAS scores of cough in the experimental group were significantly lower than that in the control group (*P* < 0.05), as shown in Fig. 4.

In the two groups, there were 2 cases of single oral painkillers (ibuprofen sustained-release capsule) in the control group and 0 cases in the experimental group. There were 2 cases of postoperative nausea in the control group and 1 case in the experimental group. There were no other adverse reactions in both groups.

## Discussion

Autologous costal cartilage transplantation as a mature technology [8], occupies an irreplaceable position in rhinoplasty. The application of autologous costal cartilage in augmentation rhinoplasty is mainly the combination of costal cartilage and prosthetic materials. The use of prosthetic materials to raise the back of the nose and the use of costal cartilage to improve the structure of the nasal tip is a more popular way in clinical use [9]. The pain of autologous costal cartilage augmentation rhinoplasty is mainly composed of nasal pain and costal cartilage area pain [3].

The pain factors of costal cartilage removal are multifactorial [10]. The most important is that the introduction of noxious stimulation after costal cartilage removal leads to postoperative pain. These stimuli include the location of surgical incision (site), nerve injury of costal cartilage, inflammatory reaction near the incision and stimulation of postoperative incision drainage tube. After removing the costal cartilage, the broken end of the incision can stimulate the corresponding segment of intercostal nerve, causing obvious pain, which is exacerbated when the patient coughs, breathes deeply and changes body position, which can lead to difficulty in sleeping, limited cough and expectoration, and even lung infection, atelectasis, intercostal neuritis or chronic neuralgia [11].

Clinically, a large number of studies have shown that when postoperative pain reaches the threshold (VAS > 3), patients are prone to anxiety and sleep disorders, which are not conducive to postoperative recovery and poor experience of surgical comfort [12–14]. In the calm state of this experiment, the VAS scores of the experimental group were lower than those of the control group at all time points. At 6 h, 24 h and 48 h after operation, the VAS scores of the experimental group were lower than that of the control group, and the difference was not statistically significant (*P* > 0.05) (VAS < 3). In this experiment at 6 h after operation, the VAS score of cough state in the experimental group was lower than that in the control group, and the difference was not statistically significant (*P* > 0.05) (VAS > 3); at 24 h, 48 h and 72 h after operation, the VAS scores of the experimental group were statistically significant (*P* < 0.05) (VAS < 3).

Clinically, the analgesic methods of autologous costal cartilage comprehensive augmentation rhinoplasty [15] mainly include opioid general anesthesia, local anesthesia at the incision and intercostal nerve block analgesia. Opioids are the standard analgesic scheme for postoperative analgesia (PCIA) [4], which has the advantages of less invasive drug delivery and systemic effects caused by intravenous medication, such as nausea and vomiting, drowsiness and excessive sedation. Local anesthesia analgesia at the incision has poor effect on long-term postoperative analgesia due to the short action time of local anesthetics. It is mostly used for intraoperative auxiliary analgesia. Intercostal nerve block analgesia can reduce systemic side effects, such as nausea, vomiting, drowsiness and dizziness. Simple intercostal nerve block analgesia can significantly reduce the pain in the costal cartilage area but cannot improve the pain in the nasal operation area.

With the increase of the numbers of autologous costal cartilage augmentation rhinoplasty, patients’ requirements for surgical safety and comfort are increasing day by day. The purpose of this study is to explore effective analgesic methods, such as intercostal nerve block in order to reduce the postoperative pain of patients with autologous costal cartilage augmentation rhinoplasty and improve the safety of operation. Intercostal nerve block is the potential space between costal pleura and intercostal muscles, allowing local anesthetic drugs to directly spread to adjacent intercostal nerves. Intercostal injection of high-dose local anesthetic drugs can spread to multiple intercostal spaces and to the nearest paravertebral space. Once entering the paravertebral space, it will spread to both sides, and block multiple intercostal nerves along this path [16].

Clinically, long-acting amide local anesthetics such as ropivacaine, levobupivacaine or bupivacaine are often used in intercostal nerve block. Because the time of action of local anesthetics is short, the addition of drugs such as dexamethasone, adrenaline and methylene blue can significantly prolong the effective time of local anesthetics. A large number of clinical trials showed that there is an incubation period of 2–4h before the anesthetic effect of methylene blue [17]. At this time, there can be burning pain around the action area; however, ropivacaine [18] has rapid onset of action and strong permeability to nerve endings, and the maintenance time is about 4–6h. The anesthetic and analgesic effects of both can just alleviate the burning pain caused when methylene blue has not played a role after injection [19]. Therefore, this study was designed to explore whether methylene blue could prolong the action time and analgesic effect of ropivacaine.

Methylene blue [5] was initially used as a stain and antidote, but in recent years some scholars have used it for postoperative analgesia in anorectal surgery and found that it has a good analgesic effect. This study found that methylene blue combined with ropivacaine in intercostal nerve block can effectively provide good postoperative analgesia for autologous costal cartilage augmentation rhinoplasty and is better than ropivacaine.

Methylene blue [20], which is strongly neurophilic can directly block the pain conduction of nerve fibers, participate in glucose metabolism, promote the continuous oxidation of pyruvate, change the acid-base balance inside and outside nerve endings and membrane potential, so as to affect nerve excitability and nerve impulse conduction; At the same time, reversible nerve demyelination can play a long-term analgesic effect without affecting its motor nerve function. The damaged nerve myelin can be completely recovered in about 30 days, during which there may be numbness in the innervated area. In this study, in order to avoid the postoperative complications of methylene blue compound solution, the analgesic concentration of methylene blue was less than 0.3%. The results show that ropivacaine intercostal nerve block combined with PCIA can effectively alleviate the early pain of patients with autologous costal cartilage syndrome augmentation rhinoplasty, but the analgesic effect of cough 24 h after operation and later is significantly weakened, and the number of cases using oral analgesics and VAS analgesic score are also significantly increased. The experimental group with intercostal block with methylene blue compound solution can achieve satisfactory postoperative analgesic effect. The VAS analgesic scores in calm and cough states were significantly reduced, and there were no cases of oral analgesics. Combined application of analgesic drugs and analgesic methods with different mechanisms not only obtains the best analgesic effect but also reduces the incidence of adverse reactions. It reduces the dosage and adverse reactions of opioids, reduces the deficiency of single analgesic mode, and achieves the effect of synergistic analgesia. Because the location of intercostal nerve block in this study coincides with the operation area of costal cartilage, this operation can be completed under direct vision, with simple and effective operation, high safety factor, less postoperative adverse reactions, and the combination of PCIA will not increase the incidence of adverse reactions.

## Conclusion

Intercostal nerve block of methylene blue combined with ropivacaine applied to autologous costal cartilage comprehensive augmentation rhinoplasty can achieve good postoperative analgesic effects, supplement the shortcomings of ropivacaine intercostal nerve block combined with PCIA, such as short postoperative analgesic time and incomplete analgesia, provide long-lasting postoperative analgesic effects and will not increase the incidence of adverse reactions. It is also conducive to the postoperative recovery of patients, improves the comfort and experience of patients, and is worthy of clinical promotion.

## Supplementary Information

Caption ESM: CONSORT—Guidelines for publishing random control trials

## Declarations

The study was discussed and approved by the hospital ethics committee, and all participants signed the ethical consent form.

## References

[1] Halepas S, Lee KC, Castiglione C (2021). Grafting in modern rhinoplasty. Oral Maxillofac Surg Clin North Am.

[2] Jenny HE, Siegel N, Yang R (2021). Safety of irradiated homologous costal cartilage graft in cleft rhinoplasty. Plast Reconstr Surg.

[3] Wee JH, Mun SJ, Na WS (2017). Autologous vs irradiated homologous costal cartilage as graft material in rhinoplasty. JAMA Facial Plast Surg.

[4] Sclafani AP, Kim M, Kjaer K (2019). Postoperative pain and analgesic requirements after septoplasty and rhinoplasty. Laryngoscope.

[5] Fransiska D, Jeo WS, Moenadjat Y (2017). Methylene blue effectiveness as local analgesic after anorectal surgery: a literature review. Adv Med.

[6] Kallewaard JW, Wintraecken VM, Geurts JW (2019). A multicenter randomized controlled trial on the efficacy of intradiscal methylene blue injection for chronic discogenic low back pain: the IMBI study. Pain.

[7] Geurts JW, Kallewaard JW, Kessels A (2015). Efficacy and cost-effectiveness of intradiscal methylene blue injection for chronic discogenic low back pain: study protocol for a randomized controlled trial. Trials.

[8] Vila PM, Jeanpierre LM, Rizzi CJ (2020). Comparison of autologous vs homologous costal cartilage grafts in dorsal augmentation rhinoplasty: a systematic review and meta-analysis. JAMA Otolaryngol Head Neck Surg.

[9] Zhao R, Pan B, Lin H (2020). Application of trans-areola approach for Costal cartilage harvest in asian rhinoplasty and comparison with traditional approach on donor-site morbidity. Aesthet Surg J.

[10] Won TB, Jin HR (2020). Complications of costal cartilage asian rhinoplasty and their management. Facial Plast Surg.

[11] Varadharajan K, Sethukumar P, Anwar M (2015). Complications associated with the use of autologous costal cartilage in rhinoplasty: a systematic review. Aesthet Surg J.

[12] Wang Y, Liu Y, Li X (2021). Prospective assessment and risk factors of sleep disturbances in total hip and knee arthroplasty based on an Enhanced Recovery After Surgery concept. Sleep Breath.

[13] Chen Z, Dong Q, Liang L (2018). Effect of different thoracic anesthesia on postoperative cough. J Thorac Dis.

[14] Ahmed A, Fawzy M, Nasr MAR (2019). Ultrasound-guided quadratus lumborum block for postoperative pain control in patients undergoing unilateral inguinal hernia repair, a comparative study between two approaches. BMC Anesthesiol.

[15] Özücer B, Dinç ME, Paltura C (2018). Association of autologous costal cartilage harvesting technique with donor-site pain in patients undergoing rhinoplasty. JAMA Facial Plast Surg.

[16] Sheets NW, Davis JW, Dirks RC (2020). Intercostal nerve block with liposomal bupivacaine vs epidural analgesia for the treatment of traumatic rib fracture. J Am Coll Surg.

[17] Lee SW, Moon SW, Park JS (2021). Methylene blue induces an analgesic effect by significantly decreasing neural firing rates and improves pain behaviors in rats. Biochem Biophys Res Commun.

[18] Anantanarayanan P, Raja DK, Kumar JN (2013). Catheter-based donor site analgesia after rib grafting: a prospective, randomized, double-blinded clinical trial comparing ropivacaine and bupivacaine. J Oral Maxillofac Surg.

[19] Roldan CJ, Nouri K, Chai T (2017). Methylene blue for the treatment of intractable pain associated with oral mucositis. Pain Pract.

[20] Roldan CJ, Chung M, Feng L (2021). Methylene blue for the treatment of intractable pain from oral mucositis related to cancer treatment: an uncontrolled cohort. J Natl Compr Canc Netw.

